# Using the Healthy Community Assessment Tool: Applicability and Adaptation in the Midwest of Western Australia

**DOI:** 10.3390/ijerph15061159

**Published:** 2018-06-02

**Authors:** Christina Tsou, Charmaine Green, Gordon Gray, Sandra Claire Thompson

**Affiliations:** 1 Western Australia Centre for Rural Health, University of Western Australia, PO Box 109, Geraldton, WA 6531, Australia; charmaine.green@uwa.edu.au (C.G.); sandra.thompson@uwa.edu.au (S.C.T.); 2 Midwest Aboriginal Organisations Alliance, Geraldton Regional Aboriginal Medical Service, Western Australia Aboriginal Advisory Council, Geraldton, WA 6531, Australia; gordongray4448@gmail.com (G.G.)

**Keywords:** interagency partnership, Aboriginal health, Australian rural and remote communities

## Abstract

Population-based studies have associated poor living conditions with the persistent disparity in the health of Aboriginal and non-Aboriginal Australians. This project assesses the applicability of the Health Community Assessment Tool and its role in improving the environment of a small community in the Midwest of Western Australia (WA). The action research cycles started with the initial reflection on the suitability of the HCAT version 2 for the local community context and whether it was fit-for-purpose. The researcher provided ‘critical companionship’, while the participants of the study were invited to be co-researchers (the Assessors) who critically examined the HCAT and assess the community. The relevant domains to the serviced town (an outer regional community) were pest control and animal management; healthy housing; food supply; community vibrancy, pride and safety; reducing environmental tobacco smoke; and promoting physical activity. The Assessors found the HCAT descriptors mostly aligned with their community context but found some of the items difficult to apply. Based on participant’s suggestions, some of the original scoring scales were reformatted. School attendance and illicit drug use were identified as a key outcome indicator for youth but were missing from the HCAT. The HCAT domains applied helped streamlining core business of agencies in the local community. The face validity of HCAT items were confirmed in this research with minor adjustments to reflect local context. Youth engagement to education is of high community concern and the development of an item would create similar interagency collaborative dialogues.

## 1. Introduction

There are significant health disparities for residents of small rural and remote communities compared to metropolitan Australian populations, particularly for Aboriginal people where the gap in life expectancy is variously reported as being between 11 and 18 years [1]. Given the complexity of life circumstances and the multi-morbidities of many Aboriginal people which make care needs more complex, partnerships between Aboriginal controlled and mainstream agencies are essential for expediting improvements in Aboriginal health outcomes [2,3,4]. There have been a number of tools developed to assess partnerships, to identify areas of strength and weakness with a view to how they might be improved [5]. While to date there have been few reports where tools have been used to assess the strength of inter-organisational partnerships between Aboriginal and mainstream organisations, quality improvement tools that focus on clinical care have been more widely adopted [6]. There are many health conditions where Aboriginal Australians suffer higher rates of disease than the general Australian population, and understanding of social determinants that exist outside of the health system has continued to grow [7]. Population-based studies have associated poor living conditions with the persistent disparity in the health of Aboriginal and non-Aboriginal Australians including the gaps in infant mortality [8], childhood infections particularly skin infections [9,10], acute persistent diarrhea [11,12], rheumatic heart disease [13]. Water supply and sanitation in remote Aboriginal communities have been identified as the priorities for health development, and the need for better quality information systems to monitor progress, equity and accountability in the delivery of water and sanitation services is considered a priority [14].

The Healthy Community Assessment Tool (HCAT) was developed and piloted through a multi-phase and iterative process that involved a series of consultations with community members and key stakeholders in the Northern Territory (NT) of Australia [15]. The tool was then trialed in remote NT Aboriginal communities, confirming its face validity with the scoring system reportedly well understood and easily followed by Aboriginal and non-Aboriginal study participants. The original authors suggested using a facilitated small group process to reduce bias in scoring of indicators [15].

The full HCAT version 2 has 13 domains with two to six items in each domain [15]. The concepts and constructs of the HCAT were informed by the socioecological theory, the Driving force, Pressure, State, Exposure, Effect, Actions (DPSEEA) framework and the Multiple Exposure Multiple Effect Model These theories and models help with assessing and understanding the relationship of diverse personal and environmental factors in human health and illness including the exploration of policy drivers that lead to pressure and change in the environment and hence health effects. Based on this understanding, actions can be taken at any point in this chain to mitigate or avoid unwanted health or social effects.

McDonald and colleagues reported on the development and trial of the HCAT in NT communities and concluded that the prototype offered many uses and benefits for community leaders, government officers and others seeking to measure environmental health conditions and address inequities in the social determinants of health in remote communities [15]. Their work focused on validating the tool constructs rather than its operation in the context of population health improvement processes. Since this initial trial, there has been no further report on application of the HCAT in Australian rural or remote communities so this paper focuses specifically on the use and adaptation of the HCAT as part of a project to improve environmental health in a Midwest community in Western Australia (WA). 

The research was undertaken as part of a broader research project which aimed at improving Aboriginal health and wellbeing and was based upon recognition that better partnerships are needed to improve service delivery in complex but under-resourced environments. We were interested in testing and using existing structured tools to support and improve the working of partnerships between Aboriginal community-controlled organizations and Mainstream organisations. Nested within efforts aimed at improving inter-organisational partnership between an alliance of Aboriginal organisations and a university centre for rural health, there was explicit interest in Aboriginal people having the opportunity to identify issues that they considered important to be researched as part of the partnership; environmental health was identified as an ongoing concern. 

This project aimed to assess the applicability and utility of the HCAT as a means of assessment. The rationale for using HCAT in this project was to use this evidence-based tool to facilitate collaborative dialogues on environmental health issues which the local interagency stakeholders saw as impacting on the health and wellbeing of the community. Priority was given to the content of the dialogues and actions coming from the discussions rather than to the absolute change in the scores for each HCAT applied.

## 2. Materials and Methods 

Five HCAT supported focus groups were conducted for data collection to inform this research, four of which occurred between April and May 2013. The first focus group involved the community-based assessors to assess the face-validity of the HCAT and to determine the stakeholder engagement strategies to implement priority action areas identified. The second focus group involved the full IA meeting membership to confirm stakeholder involvement in achieving desired improvements. Two content expert focus groups were conducted to assess both face and content validity of the tools with environmental health program teams. The final focus group took place in August 2015 when the community-based assessors reassessed the community against the HCAT items and reflect on the actions taken place since the initial assessment. 

### 2.1. Sampling and Recruitment

**Figure 1:**
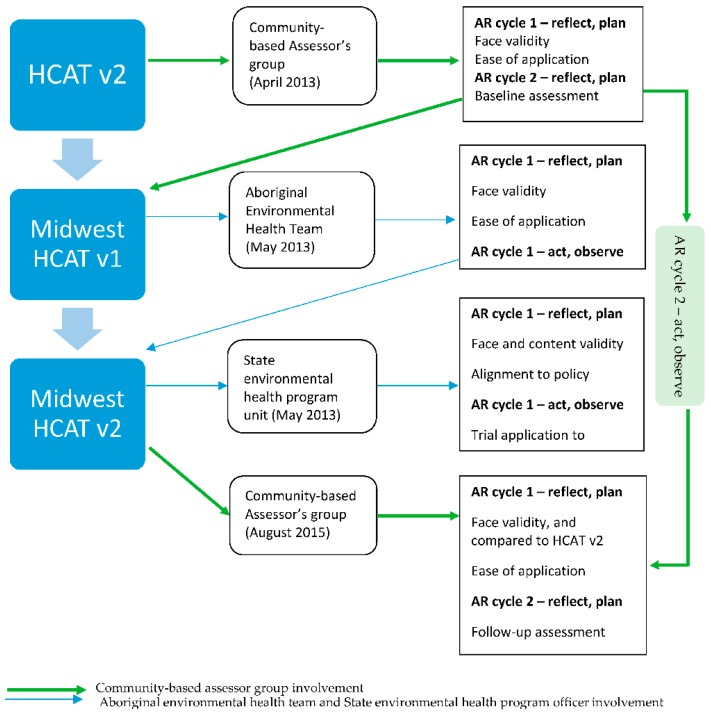
Process of validating the Healthy Community Assessment Tool version 2.

Purposive maximum variation sampling was used to ensure diversity of representation from agencies. All participants lived in the Midwest region of WA and all of whom delivered human services to this small outer regional communities. They were released by their respective agencies and volunteered to participate in the HCAT facilitated focus groups as part of their job roles. The stakeholder group involvement during the process of HCAT validation, Midwest HCAT formation, and baseline and follow-up assessments is summarized in Figure 1.

#### 2.1.1. Community-Based Assessors’ Group

Initial consultations occurred to help shape the project and understanding of community and local issues in early 2013. Thus, community-based interagency members were invited to participate in the initial baseline assessment in April 2013. Telephone invitations were followed by email information sent to a wide range of stakeholders by the local general practitioner. The research staff followed up with a face to face meeting to explain individually to stakeholders the proposed tools and its application. The council-employed community development officer based in this community saw the proposed processes as an opportunity to provide direction and evaluate the functioning of the interagency meetings (IA meetings). 

Formal invitations were also e-mailed by the local government’s district office to the broader interagency stakeholders who were residents of this outer regional West Australian location. Those who attended the first meeting formed the community-based assessors’ group (the Assessors). The Assessors assumed facilitator roles in the process of project implementation, described in detail below. 

#### 2.1.2. Midwest Aboriginal Environment Health Program Team

To compare findings in this Midwest community with other rural and remote Aboriginal communities in the Midwest region, environmental health practitioners from the Midwest Aboriginal Environment Health Program (MAEHP) were invited through the program coordinator to assess firstly the user friendliness of the tool to Aboriginal environmental health workers, and secondly the applicability of HCAT constructs to one of the remote Aboriginal communities the participants were currently working with. Participants in this group were Aboriginal men or women living in the regional centre and had more than 2 years of experience in delivering environmental health services to Aboriginal communities in the region. 

#### 2.1.3. WA Aboriginal Environmental Health Program Science and Policy Unit

The Senior Program Officer and Program Manager from the State Health Department Unit were invited to review the Midwest HCAT v2 for content validity. The state department staff ensured policy alignment and assessed whether the constructs within each item measures what they set out to measure. They then field-tested the tool on their visits to Aboriginal communities outside of the Midwest region.

### 2.2. Data Collection and Analysis

Thematic analysis was performed on contextual input received from the Assessors, and the opinions from the three groups were interpreted as a whole. All suggested revisions were incorporated and tested by having the Assessors scoring on both the original and revised versions at follow-up.

#### 2.2.1. The Action Research Process

Arising from meetings in relation to the partnership, including travel to assess environmental health in some small communities in the Midwest region, was the possibility of utilising a tool to assess the status and progress in environmental health. The action research (AR) cycles started with the initial reflection of the suitability of the HCAT version 2 for the local community context, whether it was fit-for-purpose and would support efforts of the Midwest interagency meeting to achieve local outcomes. The process involved two distinct but concurrent AR cycles: the first involved baseline assessment, reflection and modification of the HCAT v2 to ensure suitability, appropriateness and user friendliness for context; the second included baseline assessment and mapping of various action plans and community-based strategies to inform collaborative planning and tangible actions in the community. 

#### 2.2.2. Baseline Assessment—Reflect and Plan

The baseline assessment served to examine the face validity of the tool descriptors with at least 3 participants (Assessors) completing the tool for each indicator, as per instructions of the developers for administration of the HCAT. Scores were given to each component within the selected HCAT domains; overall scores were the result of consensus by Assessors and were then re-visited in discussions at a full IA meeting.

•  AR cycle 1

The Assessors were also invited to comment on the relevance of the descriptors to the Midwest context and the user friendliness of the tool. Observations were made regarding the variation in the scores given by individual Assessors and discussed the relevance and face validity of the tool constructs in each item to the local community context. The Assessors were also invited to comment both at baseline and follow-up on the appropriateness of the tool in supporting focused interagency discussions.

•  AR cycle 2

This research uses the concepts of Max Weber’s ideal type as methodological devices [16] in the understanding and analysis of interagency partnership issues in addressing environmental health needs identified by the HCAT assessment. Ideal types are concepts formulated on the basis of facts collected carefully and analytically for empirical research and are constructs or concepts used as tools in our understanding and analysis of any social issues. In this research, the concept that regional programs add capacity to local service delivery was analysed in detail by the assessors.

This AR cycle focused on identifying critical issues and achievable actions. Issues identified from the baseline HCAT assessment was mapped with other community-based action plans to cross inform findings from various community consultations. Findings from this initial assessment were presented to the full interagency meeting in order to explore collaborative potentials in addressing issues identified or implement actions suggested.

Priorities identified and potential actions were categorised into achievable actions during discussions, particularly those felt to be realisable with better coordination and a small investment of funds, and into those requiring further investigation or decision maker involvement. One or more facilitators were nominated for each achievable action areas and a timeline defined. The research process paused where the facilitators took over in the implementation phase of the action research cycle.

Full interagency stakeholders engaged in a collaborative action planning process and agreed on a process to streamline core-business and effective utilisation of resources to meet the needs of the community would look like (the ‘ideal type’). Ideally, by combining the findings from the HCAT with identified priorities in existing community-based planning documents which aimed to represent the most pressing concerns for the community, the collaborative planning discussions would be effective in identifying actions required—whether achievable with better coordination and/or a small investment of funds, or required further investigation and decision maker involvement. Facilitator(s) for each of the actions would emerge naturally, based on alignment with the core businesses of the stakeholders around the table. A community-based agency would be the primary driver of local actions and the partnership approach would help leverage support from regional programs and resources.

#### 2.2.3. Act and Observe

•  AR cycle 1

The action in this AR cycle involved adapting the tool items to ensure face and content validity of HCAT to the Midwest and WA communities. Based on the feedback at baseline, modifications were made to HCAT v2. These modifications also incorporated recommendations from a separate trial by the regional Aboriginal Environmental Health team to test the relevance of HCAT constructs to environmental health practice in Aboriginal communities in the Midwest. 

Through a facilitated process, the MAEHP team provided feedback on their experience using the tool. Revisions were made accordingly with the modified tool termed the Midwest HCAT (version 1). 

Midwest HCAT v1 was then taken to remote Aboriginal communities in Midwest and Kimberley respectively by the Senior Program Officer and Program Manager from the WA Aboriginal Environmental Health Program Science and Policy Unit for checking content validity and real-time trials in communities outside of the Midwest region. Therefore, Midwest HCAT v2 encompasses additional suggestions made based upon all three trials. 

There was opportunity in the follow-up assessment for the Midwest-based Assessors to comment on the user friendliness of the reformatted items within the relevant HCAT domains. Both the Midwest HCAT and HCAT v2 tool were provided to the Assessors in the follow-up assessment. The Assessors were requested to review both versions of HCAT instruments provided and to complete their preferred version. Refer to Figure 1 for a summary of the process of validating HCAT v2 and the formation of Midwest HCAT.

•  AR cycle 2

The action in AR cycle 2 takes the form of day-to-day business. The community-based assessors, who also delivered public services to the local community, took responsibility to facilitate the prioritized actions that was within the scope of their role functions. 

To support the continued monitoring of actions, the priority areas were used to structure the monthly IA meeting discussions. The community-based assessor group intended to focus IA meeting discussions on the issues and progress towards agreed priorities. Agenda items that fall outside of the scope of HCAT were raised as other business.

The follow-up data was collected two years from the baseline assessment. This HCAT facilitated discussion provided opportunity for the assessor group to reflect on their experience and observations over the implementation period and on the process elements of the project. This took place as part of and a focused discussion at the end of the follow-up data collection.

At each of the three assessor group meetings, the assessors reflected on whether the incorporation of the priorities generated through the HCAT application constrained the IA meeting dialogues.

#### 2.2.4. Managing Social Desirability Bias

Utilising a small facilitated group (“the Assessors”) in the healthy community assessment allowed an opportunity to discuss individual scoring and develop a consensus score for each domain assessed. The baseline scores were not revealed to the Assessors until the follow-up consensus scores were decided to reduce social desirability bias when the group scored the domains.

The group consensus scores were compared with the average scores from individual Assessors to assess alignment of individual ratings to group ratings. This comparison helped the facilitator to reflect on the degree to which ‘quiet voices’ may be lost in coming to consensus score. 

### 2.3. Role of Researchers

The researcher who initiated the research process occupied the role of focus group facilitator to provide ‘critical companionship’ [15]. The researcher constantly reflected on the risk of the structured tool restricting the scope of the interagency collaborations. This design reflects Tichen’s model of facilitation involving the key processes of observing status of the community whilst performing day-to-day duties, listening and questioning their observations, feeding back these observations, and combining challenge and support within a critical dialogue in the Assessor Group [17]. Similarly, the influence of the study process on the identification of collaborative actions and the interpretation of applicability of HCAT constructs in assessing the health of the community was also subject to regular reflection. 

### 2.4. Ethics Approval and Consent to Participate

The study was conducted as part of the More Than Talk project and approved by the Western Australian Aboriginal Health Ethics Committee (367-10/11). All participants gave informed voluntary written consent. 

### 2.5. Consent for Publication

Consent for publication was obtained through the participating agencies of research participants, and individually from all authors.

## 3. Results

### 3.1. Participant Characteristics

In total, 17 participants were included: 10 outer regional community-based Assessors, five Aboriginal environmental health program staff and two State environmental health program staff.

In the Midwest Trial, eight Assessors were involved in both the initial and baseline assessments; however, the health promotion officer was replaced by the officer for Cultural and Development officer at follow-up. There were three males and five female Assessors in both assessments. One mainstream organisation representative identified as an Australian Aboriginal descent and one participant from the local Aboriginal Community Organisation was a non-Aboriginal descent; both participants were involved in the baseline and follow-up assessments. Table 1 lists the agencies represented at baseline and follow-up. Five staff from the regional Aboriginal environmental health program participated in the trial of Midwest HCAT v1 and all of whom were local Aboriginal men and work directly with Aboriginal communities in the Midwest region. Two staff from the State environmental health unit participated in the review of the revised tool, both non-Aboriginal men regularly visited rural and remote communities in Western Australia.

### 3.2. AR 1 Cycle Findings from the Trials

The six domains deemed relevant to the town (an outer regional community) by the interagency stakeholders were pest control and animal management; healthy housing; food supply; community vibrancy, pride and safety; reducing environmental tobacco smoke; and promoting physical activity. The Assessors found the content of the descriptors mostly aligned with their community context but found that some of the items associated with healthy housing, food supply and promoting physical activity were difficult to apply. Their feedback on the items tested is summarised below.

#### Reformatting of HCAT Version 2

Modifications made to the HCAT version 2 largely represented reformatting. The participants in the Midwest Assessor group and its use by the Aboriginal Environmental Health program team found scoring the original format difficult as multiple constructs existed within the same box and were considered to have different ratings. The original scoring scales were reformatted to either a streamlined scoring scale, scoring scales with sub-headings, and in the case of laundry a checklist with weighted scales were developed. The Assessors unanimously preferred the Midwest HCAT v2 at follow-up and felt the newly formatted tool allowed a more fine-grained allocation of scores in each of the descriptor of the tool. Supplementary Table S1 summarises the modifications for selected items, and Supplementary File B for details of reasons for modification. Examples of the scale type in the Midwest HCAT are provided in the Supplementary Tables S2–S5.

### 3.3. AR2 Findings

The key observations noted in the action planning process is summarized in Table 2 below.

#### 3.3.1. Mapping of Existing Action Plans to Streamline HCAT Identified Issues and Actions

The alignment to the ‘ideal’ process was strong in the mapping phase when the alignment of local, state and federal initiatives and the potential relevance/influence of the various plans were highlighted. Feedback was then sought and received through the interagency meeting electronic mailing list and a report finalised and distributed.

#### 3.3.2. Collaborative Action Planning

Alignment to the ideal collaborative action planning process was strong when proposed actions corresponded with the core business of the participating agencies. Interagency meeting participants were also able to categorise the identified actions and the most suitable community-based facilitators for each achievable action as well as scoping relevant regional resources to support the implementation.

The collaborative planning process supported by selected HCAT helped to streamline and focus interagency discussions. However, buy-in from nominated facilitators (and relevant agencies) was heavily dependent on the relevance of identified actions to the funded core-activities of the respective agencies. For example, healthy eating and reducing environmental tobacco smoking fell within the remit of the health promotion officer, funded by the state Department of Health; healthy housing, and pest control and animal management fell within the jurisdiction of local government. Issues and actions related to community vibrancy and safety were relevant to majority of the agencies. As this domain requires multi-pronged approach and close collaboration between all agencies, the group did not identify a single lead agency and suggested that better coordination of existing activities would achieve the desired outcomes.

Alcohol and other drugs, as well as youth and education were identified as of pressing concern to the community at baseline. At the February 2014 IA meeting, the school principal requested for school attendance to be included in the project reported in this paper and a school sub-committee was formed. However, due to resource constraints, no further HCAT domains were created to address these issues and so at the time of follow-up assessment, this area was not assessed.

The discussions should have generated constructive actions; however, it was recognized that majority of the regional partnership issues identified may not have local solutions and needed to be communicated to higher level executive groups such as the Human Services Managers’ group and more senior government departments employees, not located in the regional town.

#### 3.3.3. Action/Implementation

**Figure 2:**
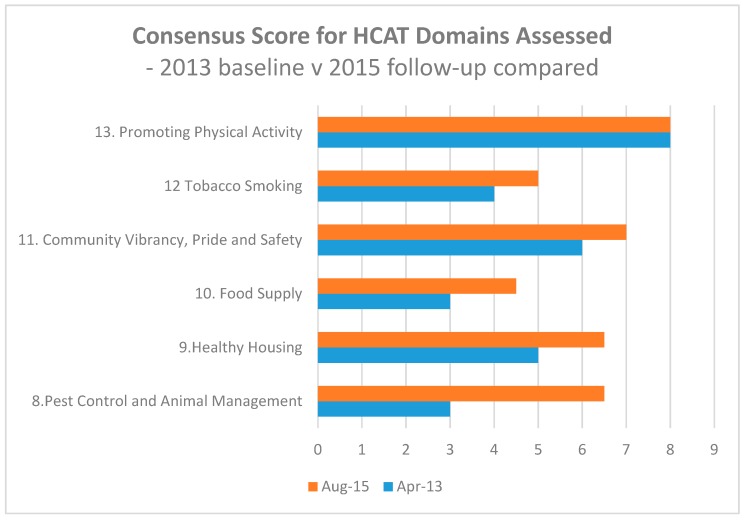
Consensus scores for HCAT domains assessed, baseline (April 2013) and follow-up (August 2015) compared.

Areas where local resources or resources were readily mobilised by the community-based facilitators included pest control and animal management, housing, healthy eating, environmental tobacco smoking, community vibrancy and safety and were associated with greater observed improvements. Figure 2 compares the April 2013 baseline and August 2015 follow-up consensus scores for the domains assessed. Where resource allocation and distribution were impacted upon by regional, state or federal level policies or programs, local stakeholders had less power to direct the course of action. For example, youth engagement was strongly linked to the employment programs, alcohol and other drugs and food supply were influenced by State and Federal policies and programmes.

In order to assess whether group consensus reflected individual assessors’ ratings for each of the domains, changes in the domain consensus score and item averages of assessors’ individual ratings from baseline to follow-up were compared. Pest control and animal management, healthy housing and tobacco smoking showed improvement in both the consensus scores and the average of individually assessed items within the domains demonstrating alignment of group consensus scores and individual assessment ratings in these domains. On the other hand, disparate views of individual assessors were observed more prominently in food supply, community vibrancy and safety, and promoting physical activity. 

#### 3.3.4. Reflection on the Collaborative Process

At the end of the follow-up assessment, the group was asked to reflect on what worked well in the HCAT application process, what didn’t work so well and what value did the process add to interagency collaborations. Finally, the group reflected on the IA partnership and how regional programs can better support local efforts.

•  What worked well?

Local ownership over the process of applying structured tool to assist assessment of the community and collaborative planning was the key strength in this experiment. Community-based interagency stakeholders were involved in the design and roll out of the project, and 5 of 8 agencies had the same person represented in the follow-up assessment. The HCAT gave a structure for the interagency meetings and the descriptors gave directions to guide the discussions.

•  What value does this process add to interagency collaboration?

Streamlining core business was identified as strength of the tool and project. Applying a structured tool with benchmarking criteria helped the IA meeting to focus collaborative discussions, align core business and identified improvements made over the two-year study period. Community-based stakeholders felt that it did not constrain the scope of IA discussions. Issues beyond the scope of HCAT were raised in open forum discussions.

•  What didn’t work so well?

Formulating common goals through IA collaboration was intended to improve buy-in from non-community-based agencies. However, despite clearly identified and agreed common goals, with the exception of health promotion (healthy food and smoking) and rangers from the local government implementing pest control programs, the buy-in from agencies based in the regional city did not change.

Where a goal aligned primarily to an outcome that was primarily seen as the domain of one agency (e.g., smoking and health) or outside of the scope of influence of the agencies (the healthiness of food sold in local retail outlets needing to consider business sustainability), agency stakeholders felt little was gained by discussing it at the IA meetings.

Despite youth and school attendance being identified in the initial assessment as a missing domain from HCAT at base-line assessment and being raised during discussion of the initial implementation as an achievable action, whole of community action which could have catalysed changes was not generated during the study period. A possible reason why school attendance fell off the IA agenda included that it was to be addressed by a dedicated committee (not incorporated into the study), but also without any criteria or domain in the assessment tool no common language was created for the IA stakeholders to communicate and put into action any agreed strategies. 

#### 3.3.5. Interagency Partnerships

Local stakeholder felt limited assistance or support from regional programs that is not based in the community. They suggested that passively waiting for referrals with inflexible service hours limited effectiveness of these regional programs. Regional services had not changed their engagement with the community over the study period with the exception of rangers who came to the community more often and attended interagency meeting as required. There was perception that regional programs were represented on the interagency meetings ‘to tick the boxes’. 

There were examples of regional services which were responsive. Of relevance to the reported activities was Homes West, the public housing agency, which undertook work that was requested and facilitated good relationships.

Regional programs were unhelpful when they had a fixed agenda instead of the much-needed hand-holding and grass-root capacity building approach. *‘They say they can do this and do that but they don’t come here and do that. So they expect us to take the whole school over there [to their head office] and educate them. So they say they can do that but nothing happens. Or they come here and tell us what to do.’* It was identified that more of ‘we can do this in [the community]’, or ‘let’s do this together’, or ‘we will run x program here for the next 10 weeks with the help of your facilitation’, and less of ‘you should do this’, ‘why don’t you do this’ would be helpful. 

Despite this, committed individuals delivering regional services were well regarded even when there was a mismatch between the regional program agenda to locally determined needs. *‘… she is genuinely interested in the safety of the community and is passionate about what she does.’*

A preconceived idea about the community from the ‘outside’ agencies was identified as barrier to engaging the local community; ‘*some of these agencies come over and still see us as the place where you have feuding … and (inaudible) … It’s a fear factor from having the lack of knowledge. An example is when the Shire amalgamated, some of those people who had never been to [the community] came for the first time for the wild flower season and they could not believe …. There were loads [of people] who come over and have no idea what [the community] has to offer. And I do wonder whether some of these agency’s preconceived ideas that [explains why] they can’t do anything to help people out here*’.

The impact of devolution policies in the education sector reflected an opposite effect on the capacity of community-based education providers. Increasingly, online resources are available with a tendency of these resources being seen to replace the face-to-face support previously provided regionally. The policy direction in public education appeared to allow for the strengths that local schools have in make a difference at the grass root; however, it underestimates the resource and capacity required to drive these changes in remote communities with scarce access to professional support. Similar to the observation of regional programs in other domains, the local school found rostered visits from Aboriginal education had a defined agenda, for example, a focus on school canteens made little difference to local needs, that is the need to support improved school attendance.

## 4. Discussion

There are a number of learnings and considerations from this initial application of the tool in practice.

### 4.1. Utility of HCAT

The HCAT contains domains assessing environmental health conditions and selected social determinants of health in small communities. Participants in this pilot agreed that using the HCAT to support interagency discussion helped to streamline core business across various public-sector functions and generated ‘useful discussions’. Our work provides some evidence that a comprehensive multi-pronged assessment tool such as HCAT can be flexibly applied in partnership with local service providers, validated by local residents, and assist to streamline inter-sectoral actions. 

The action learning approach provided a potential method for assessing progress and could support translation of findings into practice. Its utility would be improved if, based on collaborative approaches and agreed actions, there was the potential for pooled funding by which investment could be prioritised and implemented in a staged fashion for locally prioritised achievable actions. By aligning the actions needed at local, state and federal government levels, an agenda set at local level could be supported by coordination of priority initiatives. A two-year timeframe was sufficient for Midwest-based stakeholders to mobilise available resources and observe reasons for change or lack of change in relation to the HCAT descriptors. The following sub-sections discuss whether the use of an evidence-based instrument such as the HCAT is effective in meeting the ingredients for success in translating evidence into population health improvement [18].

#### 4.1.1. Overcoming Barriers by Ensuring Responsiveness of Research to Users’ Needs 

It has been argued that there is a disconnect between investigator-driven research and the priorities of communities and decision makers. This has been said to be problematic for improving population health, where change agents who can make the biggest difference in improving health behaviours and social and environmental conditions are not health professionals [18]. The application of HCAT with AR cycles reported in this paper demonstrated a promising way to overcome this commonly encountered issues in population health research translation. 

Through the processes reported in this paper, local community leaders who were generally not health professionals participated in the design of the research. Their involvement included review of the data collection instrument, analysis and interpretation of findings from the assessments. This same group of people were also the key drivers in translating research findings into change in the community. This design has an innate mechanism for the content of the research to be responsive to the users’ needs—satisfying the first ingredient for success in translating evidence into population health improvements [18].

#### 4.1.2. Focused Strategic Interagency Communication across Multiple Domains

This was not the first community assessment or action planning process to take place in the community; however, it was the first time an evidence-based tool was used to ensure comprehensive coverage of environmental health and social determinants of health domains relevant to public service delivery in the community. The process strategically facilitated interagency communication by providing common language for each domain, satisfying the fourth of Woolf et al.’s ingredient for success in translating evidence into population health improvements [18]. 

Through effective stakeholder engagement and the mapping of existing community action plans, the interactions and common issues emerged across the HCAT domains were discussed. For example, uncontrolled stray dogs in the community were identified as a contributing factor to community vibrancy and safety, and a major barrier of community residents accessing the exercise equipment available. 

Analysis of community needs based on single domains and action planning impedes the opportunity to connect single issues in different domains. However focused analysis of one of the domains can allow details exploration of an issue. The broad approach adopted by the HCAT was useful, although given limited resources available to the research, critical issues related to youth and school attendance were not fully explored. The challenge of striving for a balance between exploring a broad range of issues and having the right stakeholders around the table with enough focus and support for action was evident.

### 4.2. High Stakeholder Concern But Currently Not Assessed by the HCAT

The majority of the constructs in the HCAT represent the minimum standards for environmental conditions conducive to human health, while the domain of community vibrancy, pride and safety should ideally be tailored to the community concerns at baseline. School attendance was recognised as a major concern that would benefit from whole of community action but is not covered in any of the HCAT domain constructs. The application of HCAT in this outer regional Australian community raises the need for a construct to be created in order to facilitate similar collaborative dialogues through the ranking process and discussion.

### 4.3. Cultural and Remoteness Context of Physical Activity Requires Further Exploration

The rationale for the four open-ended questions MAEHP team participants suggested for the assessment of physical activity infrastructure requirements focused on finding out whether the needs of remote Aboriginal communities in the regions were met. The difference between the MAEHP practitioner’s opinion and the opinion of the community-based assessors may be twofold. 

First, it may be related to the size of the community. This outer-regional community is a serviced town of ~700 people, while the MAEHP provides support to remote Aboriginal communities of less than 100 people. The same physical activity infrastructure cannot feasibly be in place for communities without taking community sizes into consideration. 

Secondly and more importantly is the cultural context. The checklist of community infrastructure to promote physical activity lists the infrastructure required to promote physical activity. While the approach suggested by the MAEHP may be more culturally appropriate, understanding the physical activity needs of the community and culturally appropriate exercise. 

The infrastructure assessed by the HCAT was readily available in the community; however, it was questioned as to whether they are actually utilised, pointing to the need for further work on assessing the appropriateness of such infrastructure in meeting the physical activity needs of Aboriginal communities and that infrastructure alone is insufficient to encourage physical activity.

### 4.4. Methodology Discussions

#### 4.4.1. Social Desirability Bias Is Unavoidable in Co-Construction Projects

Detailed analysis of comparison between the group consensus scores and the averages scores from individual Assessors triggered reflections on interagency dynamics during the research process. For example, the Assessors who recently relocated to the community had very different perceptions and or assessments on the same domain to those of more longstanding community members. 

As the purpose of this paper is to report on the mechanics of applying the HCAT, its utility and how it was adapted rather than detailing the findings from the assessment, in-depth consideration of the impact of social desirability has been considered out of the scope. However, considerable disparity in the social circumstances of residents of the town inevitably impacts on an individual’s perspective of community functioning and is relevant for future application and interpretation of findings from the HCAT. 

A further source of unavoidable social desirability bias in Tichen’s model of facilitation comes from the tendency that people who initiate and are part of actions are more likely to observe positive outcomes from that particular action. This said, the HCAT facilitated focus groups were opportunities for the interagency stakeholders to reflect in a structured way on the progress towards the goals they set out to achieve at baseline. 

Considerable discussion related to the complex interaction between State-Commonwealth policy, funding, service delivery and structural changes took place, and participants were able to reflect on the grassroot impact of these layers of public sector complexity on the HCAT domains discussed in this paper. To this extent, the scope of influence of the local service providers was dependent on policy and funding decisions made outside of this community. In the case of healthy housing, local service providers had greater influence on the condition of available housing stock, saw greater possibility for actions, therefore positive outcomes or the observable changes towards desired outcomes were reported. A community-wide healthy food initiative created community dialogue but made little impact on the food stock available due to private business viability considerations. The involvement of multiple agencies and their deliberative efforts to engage with community residents may help provide a balanced perspective on issues raised in the assessment, although the extent to which this occurred cannot be ascertained.

#### 4.4.2. Transferability of Project Learnings

Partnership issues emerged from this single community assessment process was an indication of what could happen in similar communities, although such processes would be highly dependent upon the skills of the facilitator and the personalities of the participants at the table. The findings from this study would not necessarily be generalisable to other small rural communities, although similar issues could well emerge. Further observations in other communities could enable comparison of common issues across all communities. If applied nationally, there is potential to identify state variation and potentially even differentiate between federal, state and regional policy related outcomes.

### 4.5. Partnership Dynamics—Engagement of Regional Programs

Power and control have been reported as important factors in inter-organisational partnerships between Aboriginal groups and mainstream health services [19]. In any social exchange, power sets the limits and affords the possibility for action [20] Power is often driven by the movement of resources, particularly public funds [19]. Emerging themes common to health and education sector indicated rising pressure on local service providers to deliver public services while diminishing the support and hand-holding to continually develop local capacity. These experience and observation was intimately related to a centrally driven decision on movement of resources creating burden on grassroot service providers. The key to ‘getting it right’ is likely to be related to getting the power dynamic right. Instead of structuring partnership vertically, that is looking at ‘top down’ and ‘bottom up’ partnership structures [21], it may be worth considering flattening the ‘top’ and ‘bottom’ both to the same horizon of best outcome for the community. This would mean flexible and place-based considerations of resource allocation instead of a one-size fits all approach.

A positive driving force for improvement in the community was community pride in their own and others’ contribution locally. Much of the collaborative inertia [22] articulated by community-based service providers hindering effective penetration of regional programs were due to power imbalance between the two levels of programs. Feedback from the community-based service providers in this study reiterates the importance of acknowledging inherent community capability and strengths without jeopardising further improvement potentials. Improvement potentials have been barred by removing regional capacity building resources while not adding to direct investment to community-based initiatives.

### 4.6. Barriers to Collaborative Planning to Benefit Grass Root Community

Engagement of relevant stakeholders is a critical first step in collaborative planning process to satisfy Woolf et al.’s third ingredient for success in translating research into population health improvement. Although the ideal type in collaborative planning was agreed upon by all stakeholders, not all regional programs were able to fully engage during implementation. Through this research, we found the following barriers to collaborative planning to benefit this outer regional Australian community.

#### 4.6.1. Centrally Managed Program Funding Structure Continues to Create Public System Fragmentation

The International Association for Public Participation’s public participation spectrum (IAP2’s Public Participation Spectrum) defines public roles with increasing impact as being to inform, consult, involve, collaborate and empower [23]. We have learnt from this process that well-informed and deliberately consulted stakeholders genuinely wanting to work in partnership was not sufficient to guarantee commitments and collaboration from external agencies whose representatives are obliged to respond to a distant policy and program imperatives. Under the current programs’ funding structures within which majority of the regional services operate under central control, well intentioned service providers are constrained in flexibly tailoring their involvement and collaboration beyond the scope of their respective program, contributing to fragmentation within and between public sectors.

#### 4.6.2. The Grass Root Experience of Three-Tiered Governance

What do the learnings from this HCAT assessment tell us about the possibilities for genuine collaboration between grass root workers based in remote community and the levels of government responsible for the public-sector service delivery? We illustrate our learnings using examples from the HCAT domains. Local government has jurisdiction to address pest control and animal management, and to promote healthy eating, but has no control over food supply. This limits the capability of the local government to impact on food supply. For more complex issues such as housing, employment and training, support from all levels of government is essential. Improvements in healthy housing indicators were made possible through facilitation of horizontal and vertical collaborative actions. The impact of federal and state government policy changes was readily observable within this grass-root community. A prime example to illustrate the direct impact of government policy change evidenced during the study period was the multiple transitions of employment training program arrangements creating substantial disruption to the community and people living in it.

### 4.7. Study Limitations

This highlights a key limitation of the approach adopted in this study. As discussed above, this experimental process satisfied three of the four ingredients for success in translating evidence into population health improvements; however, there was no deliberate effort to ensure the research team (including the assessors) understood the broader decision-making environment at various levels of governments. A better understanding of the decision-making environment at all levels could change the strategic engagement of and communication with stakeholders. However, the dynamic within government is fluid, being heavily influenced by electoral cycles and the philosophy of those holding power. Consistent bipartisan approaches by government might allow more opportunity for local problem-solving to address the local issues faced in small remote communities. 

Our work provides some evidence that a comprehensive multi-pronged assessment tool such as HCAT could be flexibly applied in partnership with local service providers, validated by local residents, to streamline inter-sectoral actions. Its utility would be improved if based on these collaborative approaches and agreed actions, pooled funding was available to prioritise investment in a staged fashion according to locally prioritised achievable actions. By aligning actions needed at local state and federal government levels, an agenda set at local level could be supported by coordination of priority initiatives at all levels of government

Furthermore, the HCAT did not explicitly cover a critical social determinant of health—educational attainment—which could arguably be incorporated into the community vibrancy and safety domain. School attendance has been identified by the community as a key outcome indicator for youth, and a new domain could be created to provide a common language for collaborative dialogues. 

## 5. Conclusions

The HCAT contains domains assessing environmental health conditions and selected social determinant of health in small communities and the action research approach described provides a potential approach to translating research into practice. 

The face validity of HCAT items were confirmed in this research with adjustment of a few items in healthy housing, food supply and physical activity domains to reflect the local context in the formation of Midwest HCAT. The majority of the adjustments involved reformatting to allow precision in scoring across the rubric and to reduce confusion created by longer, less clearly demarcated descriptors.

Use of the HCAT domains helped support streamlining the agenda to cover core business in the local community and was key to the meaningful engagement of interagency stakeholders. The benefits of co-constructing and using action learning methods to engage local service providers throughout the research process must be balanced with the risk of social desirability bias in the healthy community assessment. Whilst the scores allocated to HCAT items provided a reasonable benchmark as to how the community had progressed toward agreed outcomes, the collaborative reflections generated were likely to be more meaningful to community-based stakeholders who work and live with the HCAT descriptors in their daily practice.

A mismatch between the needs identified by community-based service providers and regional program agenda was reported. The process documented above did not improve commitment from regional programs, with the exception of ranger services delivered from local government after amalgamation and the state Department of Housing whose core business aligned with the HCAT domains. The key reason for the lack of engagement from regional service providers was that they were administering programs with a fixed agenda and program goals that were set elsewhere, and hence were inflexible to tailoring them to the needs identified by local communities.

An instrument such as HCAT has the potential to develop into a ‘litmus test’ to gauge whether basic environmental health needs are being met. With the addition of a youth and education domain focusing on school attendance, HCAT also has the potential to concurrently provide a common language and a set of benchmarking criteria to facility evidence-based, and focused discussions on issues related to wellbeing of the local community.

Further research is indicated to examine how the HCAT (v2 or Midwest) applies to other small rural and remote communities. Using an AR methodology with comparative analysis of issues emerging and identifying achievable actions in several remote and outer-regional communities, it is likely that there would be commonality of the findings to inform regional program priorities and drive the implementation of locally-designed solutions. This will mitigate the issue of implementing centrally designed programs based on inadequate understanding of day to day realities in small remote towns.

A big question remains as to whether there is political will to innovate which results in trialing different ways in which local communities could be resourced and empowered to implement locally designed solutions. Political will would then need to be translated through various system reform initiatives and reflected in an administrative transition from a centrally driven decision making and resource allocation to allow for creative solutions which utilise the strengths within local communities and require responsiveness from external agencies to address locally defined priorities. 

## Figures and Tables

**Table 1:** Agencies contributing to HCAT assessment processes.

April 2013	August 2015	Same Person
Police	Police	No
Health—health promotion	Position discontinued (November 2014)	No
School	School	Yes
Aboriginal community controlled employment organisation	Aboriginal community controlled employment organisation	Yes
Local government (management)	Local government (management)	Yes
Local government (cultural and community development)	Local government (cultural and community development)	Yes + 1 new in 2015
Local government (youth)	Local government (youth)	No
Dept of Child Protection and Family Services	Dept of Child Protection and Family Services	Yes

**Table 2:** Alignment of the Community’s Interagency Action Planning Process to the Ideal Type Formulated at Baseline.

Action Planning	Ideal Type Formulated at Baseline	Observations	Alignment to Ideal Type
Mapping of existing community-based action plans	Found collaborative potentials to address the relevant issues identified	The mapping brought to light multiple action planning activities relevant to the community. The planning activities are led by and for the particular purposes of the lead agencies in response to federal and state government policies. These include in response to federal government policy change on employment programs, the regional investment plan developed by the state government’s regional development commission in collaboration with the DRDL outlining infrastructure priorities and the region’s 10-year blueprint for enabling economic and social development. With the amalgamation of the local government council initiated and facilitated the development of a 10-year strategic plan with the CGI.	Strong
Collaborative Action Planning—achievable actions identified	Identified action planning areas categorised into achievable with better coordination, with small investment of funds, and require further investigation and decision maker involvement.	Action areas achievable through better coordination of existing resources or small injection of funds: transport, management of domestic pets; reduce environmental tobacco smoking; food security. Action areas require more complex and longer term interventions: housing, drug and alcohol, economic development. Overwhelming evidence to support focus for more attention on addressing youth engagement, specifically around employment opportunities drawing on the strengths of local industries or created through driving an increase in retail and trade. (school attendance not explicitly identified in the initial assessment).	Strong
Collaborative Action Planning—facilitator nominated for each achievable action areas	Buy-in from nominated facilitator(s) to own the implementation phase of the action research cycle.	Healthy housing: local government led; collaborations DCP&FS, police, Department of Housing Food supply: Department of Health-led; collaborations with ACCEO, local government, Foodbank and Canteen Association, CRC, DHS. Pest control and dog management: local government district office led; collaborations with local government rangers, ACCEO (pest control training to households) Environmental tobacco smoking: Health department (community-based health promotion) led; collaborations with local government, local police, local government district office Community safety and vibrancy (including drug and alcohol): multiple agencies have core business aligned to this domain. No concrete action identified for AOD in the duration of the project.	Strong when aligned to core business
Action	Local facilitator(s) driving local actions with required support from regional programs.	Community-based interagency members took the lead in implementing actions discussed in housing, pest control and animal management, food supply and community vibrancy and safety. Increased regularity of visits by rangers, department of housing and targeted action by local member of parliament was noted. Little change in the level of contribution by other non-community-based members due to the lack of relevance of the regional program objectives to locally identified priorities. Youth employment was affected by transitions of federally funded CDEP to RJCP transitions. School attendance identified as key to improve youth engagement at follow-up and require whole of community action.	Strong when resources are easily mobilised by the community-based facilitators

* Abbreviations: DRDL = Department of Regional Development and Lands; CGI = Community Group Incorporated; DCP&FS = Department of Child Protection and Family Services; ACCEO = Aboriginal Controlled Employment Organisation; CRC = community resource centre; DHS = District High School; AOD = Alcohol and other drugs; CDEP = Community Development and Employment Program; RJCP = Regional Job and Community Program.

## Supplementary Materials

The following are available online at http://www.mdpi.com/1660-4601/15/6/1159/s1, Table S1: Modifications to the HCAT version 2, Table S2: Scoring Scale (identical to the original HCAT), Table S3: Streamlined Scoring Scale, Table S4: Streamlined Scoring Scale with Sub-headings, Table S5: Check List. Supplementary File A: Feedback and Modifications from Assessors at Baseline, Supplementary File B: The Healthy Community Assessment Tool version Midwest (MW CAT)—explanatory notes.
