# Supplementary material for: Using the Healthy Community Assessment Tool: Applicability and Adaptation in the Midwest of Western Australia
Source: Int J Environ Res Public Health. 2018 Jun 2;15(6):1159. doi: 10.3390/ijerph15061159 (PMC6024991; doi:10.3390/ijerph15061159)
Supplement: Supplementary file 1 [file ijerph-15-01159-s001.zip › Supplementary Files incl figure and tables/Figure 1.docx]

Community-based Assessor’s group

(April 2013)

**AR cycle 1 – reflect, plan**

Face validity

Ease of application

**AR cycle 2 – reflect, plan**

Baseline assessment

Aboriginal Environmental Health Team

(May 2013)

**AR cycle 1 – reflect, plan**

Face validity

Ease of application

**AR cycle 1 – act, observe**

Trial application to Aboriginal communities

State environmental health program unit (May 2013)

**AR cycle 1 – reflect, plan**

Face and content validity

Alignment to policy

**AR cycle 1 – act, observe**

Trial application to remote communities

Tool adaptation

Community-based Assessor’s group

(August 2015)

**AR cycle 1 – reflect, plan**

Face validity, and compared to HCAT v2

Ease of application

**AR cycle 2 – reflect, plan**

Follow-up assessment

* compared with baseline

* reflect on actions against agreed priorities

AR cycle 2 – act, observe

Community-based assessor group involvement

Aboriginal environmental health team and State environmental health program officer involvement

**Figure 1. Process of validating the Healthy Community Assessment Tool version 2**
