# Supplementary material for: Using the Healthy Community Assessment Tool: Applicability and Adaptation in the Midwest of Western Australia
Source: Int J Environ Res Public Health. 2018 Jun 2;15(6):1159. doi: 10.3390/ijerph15061159 (PMC6024991; doi:10.3390/ijerph15061159)
Supplement: Supplementary file 1 [file ijerph-15-01159-s001.zip › Supplementary Files incl figure and tables/Figure 2.docx]

***
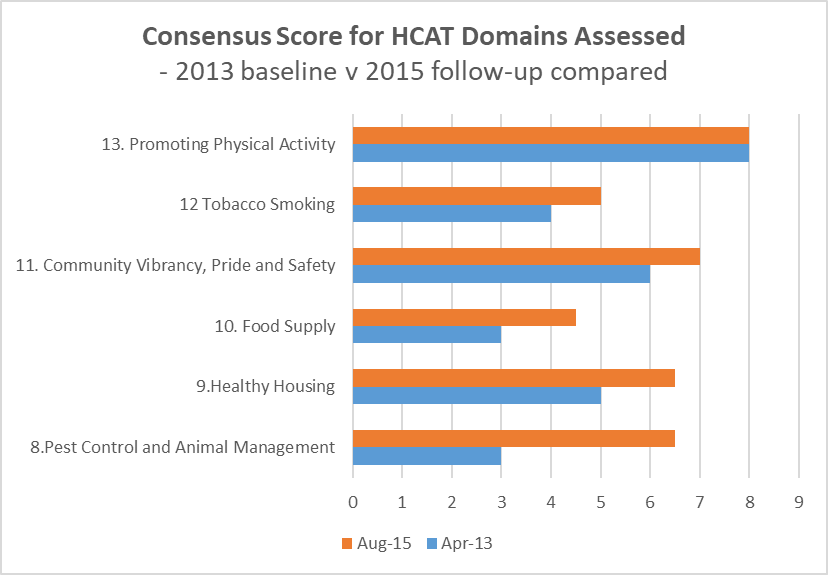
***

**Figure 2 Consensus scores for HCAT domains assessed, baseline (April 2013) and follow-up (August 2015) compared**
