# Supplementary material for: Using the Healthy Community Assessment Tool: Applicability and Adaptation in the Midwest of Western Australia
Source: Int J Environ Res Public Health. 2018 Jun 2;15(6):1159. doi: 10.3390/ijerph15061159 (PMC6024991; doi:10.3390/ijerph15061159)
Supplement: Supplementary file 1 [file ijerph-15-01159-s001.zip › Supplementary Files incl figure and tables/Supplementary Files.docx]

Supplementary File A. Feedback and Modifications from Assessors at Baseline

- **Healthy housing**

1. items modified and reformatted

The score for the laundry item (Item 9.4) was low due to absence of a community laundry. As laundry facilities were standard within the residences where people live, the Assessors believed that it was not essential for local residents to have access to a community laundry. The communal laundry facility at the caravan park was adequate to accommodate visitors staying in the community.

The Assessors found that there were multiple components in the laundry rubric making it difficult to draw a line when deciding the item scores. It was suggested that for the laundry item, there should be a weighted listing of criteria allowing individual scoring and the separation of essential and optional criteria. For instance, descriptors on community laundry would be optional in this particular community.

A staff member of the WA Aboriginal Environmental Health Program Science and Policy Unit who participated raised the concern of asbestos containing material (ACM) or inappropriately stored and disposed asbestos waste impacting on indoor air quality. Inhalation of asbestos fibres can cause asbestosis, lung cancer and mesothelioma. In the Midwest HCAT, asbestos waste disposal was added to the outdoor (ambient) air quality. It was also suggested that given the old housing stock in Midwest, asbestos/ACM in residential housing structures should to be considered under healthy housing because of its effect on indoor air quality and for ease of use in identifying, streamlining and prioritising action areas.

As a result, in the Midwest HCAT, Asbestos Containing Material (AMC) used in building was added under *Health Housing* as 9.2, and the laundry component in the original tool was reformatted as a weighted checklist and shifted to 9.4. Community laundry was listed as an optional item in the modified tool.

- **Food supply**

1. difficult to complete
2. further work required

The Assessors found this item difficult to complete. For example, availability of food scored high according to the descriptors; however, as available food is considered expensive, it is not affordable. They also found the items on food safety and promoting healthy eating were difficult to score as the conditions in Midwest did not fit neatly into any one of the boxes.

A further challenge was experienced in distinguishing availability and affordability of healthy food as the participants believed stakeholders believed mere availability would not promote healthy eating unless it was affordable. It was agreed that ‘availability of affordable healthy food’ should be assessed under one construct. It was also agreed that detailed work was required to work through the difficult-to-score items.

As food supply in the community was exclusively in the domain of private businesses, apart from limited healthy eating initiatives, the interagency stakeholders felt that their capacity to influence healthy food supply was extremely limited. Although food supply stayed as a standing agenda item during the two-year trial period, limited discussion was generated beyond the initial assessment. This item was eventually taken off the agenda but participants agreed to review this item in future.

- **Promoting Physical Activity**

1. inconsistencies in HCAT v2 rectified
2. new item introduced as an alternative option for small remote Aboriginal community without physical activity infrastructure

Participants found it confusing that adults aged ≥65 years were a category on the scoring sheet but were not being identified in the HCAT rubric. Furthermore, in a rural and remote context, particularly in Aboriginal communities, participants felt that the concept of physical activity would be different to the mainstream conceptualisation particularly for Aboriginal people over the age of 45 years, and especially over 50 years.

The MAEHP team participants suggested replacing the checklist of community infrastructure to promote physical activity with four open-ended questions to align with the needs of other remote Aboriginal communities in the region. These questions were

1. What cultural exercises do you do to keep you healthy?
2. What exercise / sporting activities do you do to keep healthy?
3. What exercise / sporting activities would you want to do for your own health?
4. What other activities would make your health better?

Interestingly, when explored with the wider group of the Midwest Assessors, they considered the original physical activity items more relevant to the context of this particular community.

The four open-ended questions were retained as an alternative to the checklists in the Midwest HCAT v2.

- **Community Vibrancy, Pride and Safety**

1. reformatting
2. new constructs added on local governance
3. missing item identified

Similar to the laundry item, the Assessors found items in this domain difficult to score due to the presence of multiple constructs within each cell in the rubric. Reformatting options were suggested and based on the suggestion, the streamlined scoring scale with headings (see Table 4 below) was developed.

‘Strong leadership and local coordination possible to create change in the community’ was suggested to be pertinent to excellence in community vibrancy. To qualify as good vibrancy, leadership and local coordination make it possible to steer community-based programs. The Assessors felt that when the leadership and local coordination for basic level of service delivery is present. When local coordination of service delivery is not possible, community vibrancy would be poor or very poor. This construct was added to item 11.1 – the vibrancy rubric.

The HCAT did not explicitly cover a critical social determinant of health – participation in education - which the community-based Assessors believed was related to the attainment of community vibrancy and safety outcomes.

Illicit drugs such as marijuana and methamphetamine were also raised by both groups in the Midwest Trials as of high concern to the community, but not issues considered within the HCAT.

Supplementary File B. The Healthy Community Assessment Tool version Midwest (MW CAT) – explanatory notes

The Western Australia Centre for Rural Health (at the time of data collection the Combined Universities Centre for Rural Health) conducted the WA pilot of the original Menzies HCAT first took place in Mullewa with six interagency stakeholders who examined the face validity and suggested adjustments to wording and formats of the items used (the last six items from the original tool). This initial WA Trial took place as part of ‘Building the Mullewa Model’ case study.

Based on the feedback from the Mullewa Trial, the full tool was examined in detail and where required components were reformatted.

The original and reformatted components were trialed with Environmental Health Practitioners of different levels of EH Experience in the Midwest Aboriginal Environmental Health Program (MAEHP) to test for relevance of HCAT constructs to Environmental Health practice in Aboriginal Communities in the Midwest.(The Bundiyarra Trial). MAEHP team members have provided valuable feedback based on their practical experience and revisions made accordingly to form The Healthy Community Assessment Tool Midwest version 1 or The Midwest Healthy Community Assessment Tool version 1 (MW HCAT v1). ***Added constructs are bold and italicized.***

MW HCAT v1 was taken to remote Aboriginal communities in Midwest and Kimberley respectively by the Senior Program Officer and Program Manager, *WA Aboriginal Environmental Health Program Science & Policy Unit* for real time trials. MW HCAT v2 encompasses suggestions provided from these trials and the second Bundiyarra Trial.

The MW HCAT case study is funded by Healthway through the More Than Talk research translation into practice grant.

**HCAT Item Brief**

1. **Water Supply**

The objective of a water supply system is to provide adequate water that is suitable and safe for a variety of uses (including for consumption, domestic needs, personal hygiene, bathing and food preparation) in the community. A system includes storage and treatment facilities and the distribution system. The quantity of the water available and the microbiological, chemical and radiological aspects and other disease-causing agents that can affect water are of major importance to health outcomes.

Drinking water quality is measured against Australian Drinking Water Guidelines (ADWG) values for physical, chemical, microbiological characteristics. The volume of water required by a community is measured against ADWG for quantity (that is, 1000-1200 litres per person per day).

To help assess your community’s water needs refer to the NHMRC *Community Water Planner – A tool for small communities to develop drinking water management plans* to assess their community’s water supply needs and develop a water management plan.

1. **Sewerage System**

The objective of a sewerage system is to transport sewerage or effluent from properties to the treatment plant for treatment and final disposal, addressing public health and environmental concerns and in accordance with current legislation.

A sewerage system is usually a network of pipes through which sewage is transported to a centralised sewage treatment plant. Here, primary treatment (screening, grit removal, primary settling), secondary/biological treatment (aerobic oxidation, settling) and tertiary treatment (nitrification) takes place. Remote Indigenous communities and small rural towns may have septic tanks with on-site effluent disposal (e.g. soakage trenches, leach drains or wells) on individual properties, septic tank effluent disposal (STED) systems (where primary treatment takes place in septic tanks on individual properties and septic tank effluent is then collected through a network of pipes and treated centrally) or may have a fully reticulated sewerage system.

Final effluent disposal is disposal of the treated wastewater through evaporation, discharge or reuse (such as irrigation). Excellent final effluent disposal is via reuse, with no contamination of water supplies or the natural environment, and all health department and environmental requirements are met.

1. **Air Quality**

Polluting emissions that reduce the air quality generally occur from 3 sectors: transport, residential and industry. Residential sources include heating with wood, backyard burning and domestic appliances. In rural and remote areas the smoke from burning garden waste or household refuse, back burning and wild bush fires can cause some people to have serious respiratory problems.

Air pollution occurs when the air contains gases, fumes or odour in harmful amounts (that is, amounts which could be harmful to health or comfort of animals or which could cause damage to plants and materials). Pollutants can include carbon monoxide from car exhausts, pesticides used in aerial weed control and sulphur dioxide from combustion of coal. There are also natural sources such as windblown dust and smoke from bush fires.

Indoor air is air within a building that occupied for at least one hour a day including the office, classroom, home and store. Indoor air quality can be defined as the totality of attributes of indoor air that affect a person’s health and wellbeing. Sources of indoor air pollution include tobacco smoke, gas cookers, unflued gas heaters, fungi, microbial contamination, house dust mites, particulates and air toxins such as formaldehyde.

Asbestos containing material (ACM) or inappropriately stored and disposed asbestos waste is of concern to indoor air quality. Inhalation of asbestos fibres can cause asbestosis, lung cancer and mesothelioma. There are two main types of asbestos, ‘friable’ or ‘non-friable’. Friable asbestos can be easily crumbled, pulverised or reduced to powder by hand pressure when dry. ‘Non-friable asbestos is any ACM other than friable asbestos these include, but not limited to, asbestos cement building products, vinyl floor tiles, friction materials, and any product where the asbestos is locked into the matrix. In the Midwest HCAT, asbestos waste disposal is added to the outdoor (ambient) air quality, while Asbestos or ACM in residential housing structures influencing indoor air quality is moved to healthy housing for ease of use in identifying, streamlining and prioritising action areas.

1. **Public Toilets**

A public toilet is a toilet that the general public has free access to, regardless of payment/non-payment. The general public is free to access the toilet without having to be a resident or have some other link to the community.

A good public toilet design takes into account visibility, access, natural light, proximity to other facilities, building design and orientation, landscaping, building materials and finishes, management, maintenance and security.

Public toilets are crucial infrastructure necessary for the physical comfort and social well-being and practical operation of a functioning community. The provision of public toilets has implications for public and individual health, transport, crime prevention, urban design, economic and cultural development and social equity.

As a guide, public toilets should be available whenever approximately 100 patrons or spectators normally attend an activity centre, while a reduced number applies at other places where people gather, for example, when public barbeque facilities are a focal point for people to gather.

1. **Solid Waste Disposal**

Regular organised rubbish disposal is an important factor in community health. It can prevent chemical and food poisoning, physical trauma from sharp and insecure objects and infectious diseases caused by vermin and insects.

Solid waste is produced by all sectors of society and includes household waste such as garbage and garden clippings, other waste such as street bins and street sweepings and wastes from retail, commercial and industrial activities. Putrescible materials comprise the largest proportion of municipal waste, with paper/wood products, glass, metals and plastics constituting a significant proportion. Solid waste may be disposed by recycling, composting, incineration, landfill and on-site landfill. Solid waste management is an environmental problem with implications for land, water and air quality, environmental health and land use.

Here we refer to the delivery system as the processes that occur from house to tip, including waste collection vehicle(s), waste transfer stations and landfill site, also the level of services provided to households and public areas.

1. **Community Drainage, Roads and Footpaths**

Flooding has been described as situations in which ‘watercourses overflow and inundate either part or all sections of the community’. Ponding has been described as the formation of ‘pools of still water that remain stagnant for a period of one week or more and cover an area of at least 10 square metres’. Flooding and ponding can result from rain events and other surface runoff. It can result in problems for the community including sanitation system failures, damaged electricity supplies, excessive mould growth, inaccessible roads and breeding of disease carrying mosquitoes. Risks to human health include an increased risk of death and injury, faecal-oral transmitted disease, vector borne disease, rodent borne disease and mental health problems. Contact with flood water should be minimised (including preventing children from playing in floodwater) in order to avoid waterborne illness such as diarrhoeal disease.

Risk factors specific to rural and remote Australian roads include: greater distances travelled, higher speed limits, poorer road quality and unsealed roads, increased diversity in types of vehicles, and delays in retrieval and accessing medical treatment and rehabilitation. There is a lower risk of collision with other vehicles but higher risk of collision with livestock and wildlife. Police enforcement of speed limits, alcohol use and seatbelt wearing has been found to be less on rural roads than in urban areas. Safe roads require traffic management, signs and markings, maintenance and pedestrian and bicycle safety features to ensure risk of injury is minimised. The road surface is vital for road safety as it helps vehicle tyres grip the road. Dust from unsealed roads and other community areas can affect health and increase wear and tear on vehicles and equipment.

Footpaths are public spaces. Well designed and maintained footpaths provide safe walking and access conditions and promote the use of public spaces and physical activity. Their primary role is to provide access for all people to move about with safely and without obstruction. The availability of footpaths on streets where children live can reduce levels of child pedestrian trauma. Smooth footpath surfaces ease wheelchair travel, aid parents with prams, and assist people using walking frames. The provision of tactile sections of the footpaths will assist people who are vision impaired.

An additional component, 6.4, on Flood Protection has been suggested by the Midwest Aboriginal Environmental Health Practitioners to specify and reinforce the importance of embankment in flood prone communities.

1. **Electricity Supply System**

The term electrical power supplies include the power distribution system as well as the primary or secondary sources, including conversion, batteries, chemical fuel cells, wind and solar power and generators.

Access to electricity and gas allows for the operation of health-related infrastructure, such as lighting, heating and cooling, water heating, refrigeration of foods, power supply for kitchen appliances, communication, education and the use of other electrical equipment. The electricity supply is required to be consistent in the voltage of power delivered so as not to damage essential household items such as refrigerators.

Some communities may not have access to an organised electricity supply and where a supply is provided, not all houses may be connected. The cost of electricity may prohibit householders having a continuous supply of electricity, especially in communities where the majority of community members have low levels of income. In this situation problems may arise as it concerns storing food safely, doing laundry and in some contexts maintaining good personal hygiene.

1. **Pest Control and Animal Management**

Pest control is the reduction or regulation of the population of noxious, destructive or dangerous insects or other animals through various means, including chemical and biological processes. Vermin refers to various small animals or insects, such as rats or cockroaches that are destructive, annoying or injurious to health.

Animal management can be defined as the act, manner or practice of managing; handling or control of animals.

Good animal management practice aims to promote and facilitate responsible ownership of dogs and cats, animal welfare and the benefits of owning a companion animal. It aims to reduce the risks posed to human health from animals including disease and injury. Strategies to manage domestic animals include their identification and registration; collecting and impounding stray animals; promoting the health and welfare of animals; restricting the number and type of animals in a community; and taking action against dangerous or diseased animals. Animal Management Acts or Model Codes of Practice exist in many States to guide animal management planning, monitoring and action.

The term domestic animal refers to domesticated animals that live in physical proximity to humans, such as cats and dogs (pets) and guard animals or even food species (pigs) kept very close, for example, to live on domestic food scraps and/or their body heat can be used as ‘stable heating’. In some communities, native animals such as kangaroos are reared and kept as domestic pets.

Livestock refers to domestic animals (such as chickens, pigs, horses, cattle) intentionally reared in an agricultural setting to produce food or fibre, or for its labour.

Vermin refers to small animals, insects and mites that have the potential to spread disease or cause injury to humans and/or cause damage to infrastructure, for example, cockroaches, rats, mice, fleas and scabies.

A holistic approach to animal management in remote Indigenous communities recognises cultural and spiritual aspects, the Indigenous perspective that dogs are integral to the fabric of remote communities, and that the health of the dogs is intrinsically linked to the health and well-being of the community.

1. **Healthy Housing**

Poor housing and poor living conditions promote the spread of infectious diseases and place at risk the social and emotional wellbeing of those who live in the house and the wider community.

Health hardware is the term used to describe the physical equipment necessary for healthy, hygienic living. The equipment must have design and installation characteristics that allow it to function and to maintain or improve health status, e.g. to be able to wash there needs to be a reliable supply and sufficient quantity of water available, efficient and functional plumbing systems at community and household levels, while items such as the shower rose, taps and drain need to be of appropriate technology and functional. Only some key aspects of housing that directly impact on preventing infections are included here.

In the Midwest HCAT, Asbestos Containing Material (AMC) use in Building has been added to this item as 9.2 and the laundry component in the original tool reformatted and shifted to 9.4.

1. **Food Supply**

Food and nutrition are important aspects of health and wellbeing. Poor nutrition due to insufficient, low quality or unreliable food intake leads to ill health. It also contributes to existing inequities in health because inadequate or poor-quality food intakes are most commonly experienced by people with the worst social and economic status and other forms of individual or environmental disadvantage. In remote and rural areas, food and nutrition must be viewed in the context of the food supply, its availability, freshness, cleanliness and year-round variety. The term ‘food supply’ is used in this report to refer to those aspects of the supply of food in a community that affect the food security of individuals, households or an entire population. These aspects of the food supply include the location of food outlets (retail and prepared foods) within a community; the availability of food within those stores; the price, quality and variety of the food that is available; management of the store; and the way that different foods are identified and promoted

The term food security refers to the ability of individuals, households and communities to acquire appropriate and nutritious food on a regular and reliable basis using socially acceptable means. Food security is determined by the food available in a community (food outlet, market garden, home grown, proximity to a major centre) and whether people have adequate resources and the skills needed to acquire and use (access) that food. People who are socio-economically disadvantaged experience food insecurity, for example, the unemployed, low wage earners, single mothers and in some Indigenous communities.

The term food outlet includes shops such as supermarkets that sell food that generally needs to be prepared and ‘take-away’ stores that largely sell pre-cooked or pre-prepared food.

In MW HCAT, ‘availability of healthy food’ is replaced by ‘availability of affordable healthy food’ because the Mullewa based stakeholders believed merely availability does not promote healthy eating unless it is affordable.

1. **Community Vibrancy, Pride and Safety**

Community vibrancy, pride and safety are linked to increased physical and mental health, sporting and academic achievement, local economic development, and lower rates of homicide, suicide, and substance abuse. The vibrancy, pride, feel and safety of a community can also have economic and environmental benefits. Communities that are well maintained and appealing increase property values attract tourism and promote retail sales.

Vibrancy refers to the opportunities within the community for cultural and artistic expression and participation and for cultural values to be expressed through the arts, promoting a culturally relevant visual and auditory environment. Arts and culture in a community relate to health and safety outcomes because they promote healing, physical activity, social connections and community engagement. Artistic outlets such as gardens, murals and music support a healing environment. Artistic expression, such as dance, can encourage physical activity and create environments that engage youth, resulting in positive behaviour outcomes.

Pride has been defined as a sense of one’s own worth. Community pride builds the community’s identity, self-esteem and self-respect, and ultimately builds the community itself. A welcoming, well-maintained, clean and culturally appropriate appearance of a community can encourage people to go out, fostering social connections and encouraging physical activity.

The feel and safety of a community relates to health and safety outcomes because it can affect people’s willingness and ability to engage in physical activity, interact socially, and reinvest in their community. The feel and safety can contribute to a general increase in community networks and trust by creating a community feel through which people are encouraged to interact with each other in a safe environment.

The MAEHP staff members have experienced firsthand the importance of leadership and local coordination to the successful delivery of environmental health program to remote and rural Aboriginal communities. To capture this in the assessment, an additional construct is added to component 11.1 under the sub-heading of Local Governance.

1. **Reducing Environmental Tobacco Smoke**

Second-hand cigarette smoke has been linked with a range of serious and life-threatening health impacts including heart disease, cancer, asthma and other respiratory problems. While most evidence relates to indoor exposure, there is emerging evidence on how smoking affects air quality in outdoor locations such as playgrounds.

Children exposed to second-hand smoke are at an increased risk of asthma, sudden infant death syndrome (SIDS), acute respiratory infection and ear problems.

Smoking bans support smokers to reduce cigarette consumption and increase rates of quitting.

Although inhaled illicit drugs such as Marijuana, Gunja are raised by both groups involved in the Midwest Trials, it is considered a separate issue to tobacco smoke.

1. **Promoting Physical Activity**

Two types of physical activity are discussed here:

- - - Incidental exercise. This is the movement people perform when undertaking daily activities, such as walking/cycling to work or walking the dog. Incidental exercise is promoted by the presence of footpaths, cycle paths, etc.
    - Structured exercise. This is planned and organised physical activity such as playing sport, weight training or attending aerobics classes.

Opportunities for both incidental and structured exercise should be available and promoted in a community.

Healthy eating and physical activity are the cornerstones of good health and are particularly important for the healthy growth and development in children. Engaging regularly in moderate to large amounts of physical activity is seen to be protective against unhealthy weight gain that can lead to developing chronic disease in adulthood. Regular physical activity, active play and sports can be a practical means to achieving numerous health gains, either directly or indirectly, though its positive impact on other major risks, in particular high blood pressure, high cholesterol and obesity. In addition, it promotes general psychosocial well-being, reduces stress, anxiety and depression, improves bone health and helps control weight gain. Low levels of physical activity during childhood have been linked with risk factors for cardiovascular disease and diabetes in adulthood. Those engaged in sedentary occupations and inactive recreations, such as watching television, are at high risk of unhealthy weight gain.

Engaging in regular physical activity offers benefits across all age ranges. Physical activity is important for healthy aging, improving and maintaining quality of life and promoting and maintaining independence. Children and young people, women, the elderly and people with disabilities have varying exercise needs. For the general adult population, the greatest health benefit is gained by performing at least 30 minutes of cumulative moderate physical activity every day (or at least 5 days per week). This level of activity can be reached through a broad range of appropriate and enjoyable physical activities in people’s daily lives, such as walking to work, climbing stairs, gardening, dancing, as well as a variety of other leisure and recreational sports.

Children need a minimum of 60 minutes of moderate to vigorous physical activity every day. Television viewing of more than two hours a day in childhood and adolescence is associated with poor fitness, smoking, raised cholesterol and being overweight in adulthood. Physical activity is very important for children between 5 and 12 years of age. It benefits them by promoting healthy growth and development; build strong bones and muscles; improve balance and other skills; maintain and develop flexibility; help achieve and maintain a healthy weight; improve cardiovascular fitness; help relaxation; improve posture; provide opportunities to develop friendships; and improve self-esteem. As important, it promotes the healthy habit of regularly engaging in physical activity to carry throughout life.

The Check list of community infrastructure to promote physical activity is replaced by four open-ended questions to suit the needs of the Aboriginal communities in this region at this stage of their development.

**Supplementary Table 1: Modifications to the HCAT version 2**

| **Items** | **Midwest HCAT Components** | **Scale Type in Midwest HCAT** | **Compare to Menzies HCAT version 2.0** |
| --- | --- | --- | --- |
| Table 8 Pest Control and Animal Management | 8.1 Domestic Pets | Scoring Scale | Identical |
|  | 8.2 Livestock | Scoring Scale | Identical |
|  | 8.3 Vermin | Scoring Scale | Identical |
| Table 9 Healthy Housing | 9.1 Personal Hygiene | Streamline Scoring Scale with Sub-headings | Reformatted |
|  | 9.2 Asbestos Containing Material (ACM) use in Buildings | Scoring Scale | **Moved from construct within 3.2** |
|  | 9.3 Healthy Food Storage and Preparation | Streamlined Scoring Scale with Sub-headings | Change to 9.3 The Ability to Safety Store and Prepare Healthy Food |
|  | 9.4 Laundry | Check List | 9.2 Laundry |
| Table 10 Food Supply | 10.1 Food Outlet Infrastructure | Scoring Scale | Identical |
|  | 10.2 Access to Food Outlet | Scoring Scale | Identical |
|  | 10.3 Availability of Affordable Healthy Food | Scoring Scale | The word 'affordable' added to qualify healthy food |
|  | 10.4 Affordable Healthy Food | Scoring Scale | Identical |
|  | 10.5 Food Safety | Scoring Scale | Identical |
|  | 10.6 Promoting Healthy Eating | Scoring Scale | Identical |
| Table 11 Community Vibrancy, Pride and Safety | 11.1 Vibrancy | Streamline Scoring Scale with Sub-headings | Reformatted |
|  | 11.2 Pride | Streamline Scoring Scale with Sub-headings | Reformatted |
|  | 11.3 Safety | Streamline Scoring Scale with Sub-headings | Reformatted |
| Table 12 Reducing Environmental Tobacco Smoke | 12.1 Private Space (eg house, car) | Scoring Scale | Identical |
|  | 12.2 Public Space (eg in and around public buildings and facilities (store, health centre, school etc) | Scoring Scale | Identical |
| Table 13 Promoting Physical Activity | 13.1 Children under 5 yrs | Scoring Scale | Identical |
|  | 13.2 Physical Activity for 5 to 18 yrs | Scoring Scale | Identical |
|  | 13.3 Physical Activity for 18 to 45 yrs | Scoring Scale | Identical |
|  | 13.4 Physical Activity for 45 yrs+ | Scoring Scale | Identical |
|  | 13.5 Gender Equity | Scoring Scale | Identical |
|  | 13.6 Facilities to Promote Physical Activity - Please tell us your understanding of the following | A set of four open-ended questions | Provide open-ended question as alternative to Table 14 if checklist considered irrelevant to local community context. |

**Supplementary Table 2: Scoring Scale (identical to the original HCAT)**

**Supplementary Table 3: Streamlined Scoring Scale**

**
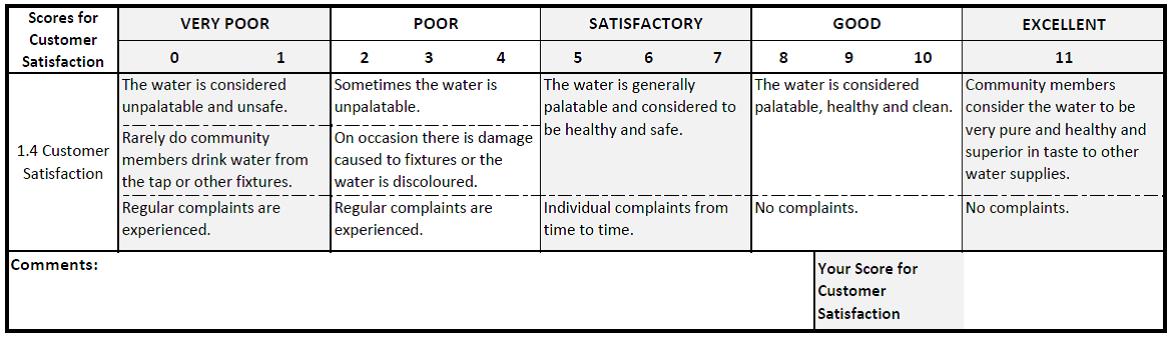
**

**Supplementary Table 4: Streamlined Scoring Scale with Sub-headings**

**Supplementary Table 5: Check List**
