# Supplementary material for: Using the Healthy Community Assessment Tool: Applicability and Adaptation in the Midwest of Western Australia
Source: Int J Environ Res Public Health. 2018 Jun 2;15(6):1159. doi: 10.3390/ijerph15061159 (PMC6024991; doi:10.3390/ijerph15061159)
Supplement: Supplementary file 1 [file ijerph-15-01159-s001.zip › Supplementary Files incl figure and tables/Table 1.docx]

**Table 1: Agencies contributing to HCAT assessment processes**

| **April 2013** | **August 2015** | **Same Person** |
| --- | --- | --- |
| Police | Police | No |
| Health – health promotion | Position discontinued (Nov 2014) | No |
| School | School | Yes |
| Aboriginal community controlled employment organisation | Aboriginal community controlled employment organisation | Yes |
| Local government (management) | Local government (management) | Yes |
| Local government (cultural and community development) | Local government (cultural and community development) | Yes + 1 new in 2015 |
| Local government (youth) | Local government (youth) | No |
| Dept of Child Protection and Family Services | Dept of Child Protection and Family Services | Yes |
