# Supplementary material for: Using the Healthy Community Assessment Tool: Applicability and Adaptation in the Midwest of Western Australia
Source: Int J Environ Res Public Health. 2018 Jun 2;15(6):1159. doi: 10.3390/ijerph15061159 (PMC6024991; doi:10.3390/ijerph15061159)
Supplement: Supplementary file 1 [file ijerph-15-01159-s001.zip › Supplementary Files incl figure and tables/Table 2.docx]

**Table 2 Alignment of the MW Interagency Action Planning Process to the Ideal Type Formulated at Baseline**

| Action Planning | Ideal Type | Observations | Alignment to Ideal Type |
| --- | --- | --- | --- |
| Mapping of existing community-based action plans | Found collaborative potentials to address the relevant issues identified | The mapping brought to light multiple action planning activities relevant to the community. The planning activities are led by and for the particular purposes of the lead agencies in response to federal and state government policies. These include in response to federal government policy change on employment programs, the regional investment plan developed by the state government's regional development commission in collaboration with the DRDL outlining infrastructure priorities and the region's 10-year blueprint for enabling economic and social development. With the amalgamation of the local government council initiated and facilitated the development of a 10-year strategic plan with the CGI. | Strong |
| Collaborative Action Planning - achievable actions identified | Identified action planning areas categorised into achievable with better coordination, with small investment of funds, and require further investigation and decision maker involvement. | Action areas achievable through better coordination of existing resources or small injection of funds: transport, management of domestic pets; reduce environmental tobacco smoking; food security. Action areas require more complex and longer term interventions: housing, drug and alcohol, economic development. Overwhelming evidence to support focus for more attention on addressing youth engagement, specifically around employment opportunities drawing on the strengths of local industries or created through driving an increase in retail and trade. (school attendance not explicitly identified in the initial assessment). | Strong |
| Collaborative Action Planning - facilitator nominated for each achievable action areas | Buy-in from nominated facilitator(s) to own the implementation phase of the action research cycle. | Healthy housing: local government led; collaborations DCP&FS, police, Department of Housing Food supply: Department of Health-led; collaborations with ACCEO, local government, Foodbank and Canteen Association, CRC, DHS. Pest control and dog management: local government district office led; collaborations with local government rangers, ACCEO (pest control training to households) Environmental tobacco smoking: Health department (community-based health promotion) led; collaborations with local government, local police, local government district office Community safety and vibrancy (including drug and alcohol): multiple agencies have core business aligned to this domain. No concrete action identified for AOD in the duration of the project. | Strong when aligned to core business |
| Action | Local facilitator(s) driving local actions with required support from regional programs. | Community-based interagency members took the lead in implementing actions discussed in housing, pest control and animal management, food supply and community vibrancy and safety.  Increased regularity of visits by rangers, department of housing and targeted action by local member of parliament was noted. Little change in the level of contribution by other non-community-based members due to the lack of relevance of the regional program objectives to locally identified priorities. Youth employment was affected by transitions of federally funded CDEP to RJCP transitions. School attendance identified as key to improve youth engagement at follow-up and require whole of community action. | Strong when resources are easily mobilised by the community-based facilitators |

*Abbreviations: DRDL = Department of Regional Development and Lands; CGI = Community Group Incorporated; DCP&FS = Department of Child Protection and Family Services; ACCEO = Aboriginal Controlled Employment Organisation; CRC = community resource centre; DHS = District High School; AOD = Alcohol and other drugs; CDEP = Community Development and Employment Programme; RJCP = Regional Job and Community Programme.
